# Microbiome complexity shapes metabolism

**DOI:** 10.1371/journal.pbio.3001793

**Published:** 2022-09-21

**Authors:** Lev Litichevskiy, Christoph A. Thaiss

**Affiliations:** Microbiology Department, Institute for Immunology, and Institute for Diabetes, Obesity, and Metabolism, Perelman School of Medicine, University of Pennsylvania, Philadelphia, United States of America

## Abstract

How does the metabolism of mice with a minimal microbial community compare to that of germ-free and conventionally colonized mice? This Primer explores a study in PLOS Biology which uses a novel isolator-housed metabolic cage system to find out.

## Teaser

Equipped with a novel isolator-housed metabolic cage system, a study in *PLOS Biology* assessed how the metabolism of mice harboring a defined minimal microbial community (OligoMM12) differs from that of germ-free and conventionally colonized mice.

**Fig 1:**
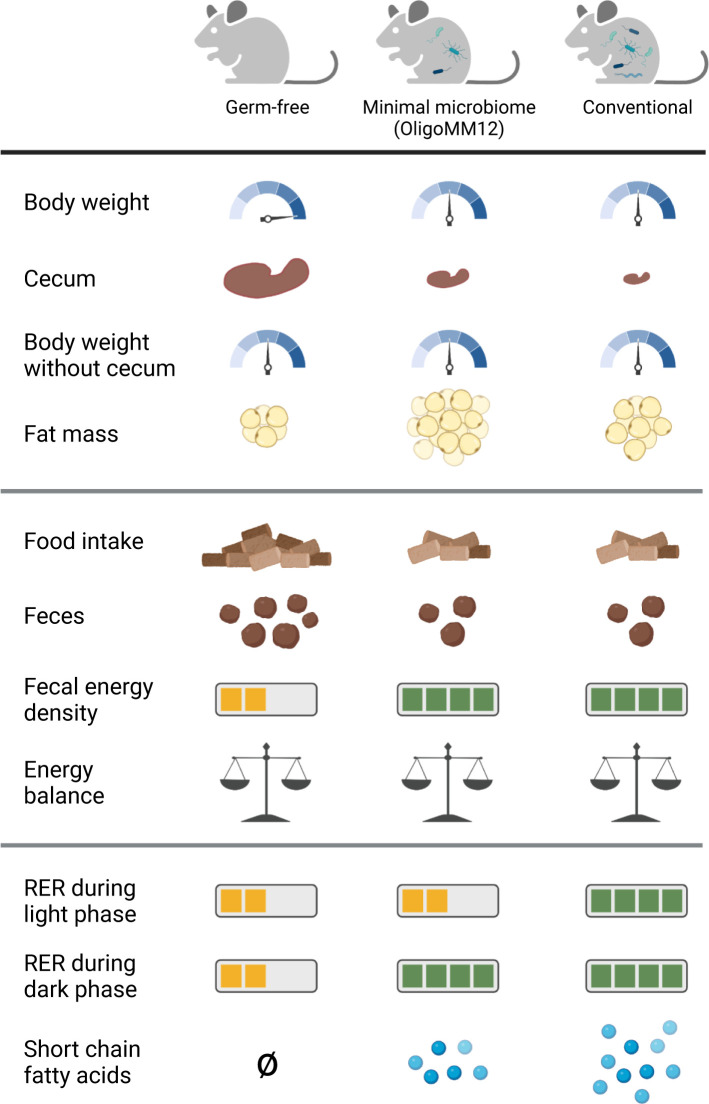
Summary of key metabolic parameters in mice harboring microbiomes of different complexity. (Top) GF mice are heavier than OligoMM12 mice and conventional mice, but after subtracting the weight of the cecum, all 3 groups have approximately the same weight. OligoMM12 have greater fat mass than the other 2 groups. (Middle) GF mice have greater food intake, increased fecal output, and lower fecal energy density than the other 2 groups. The result is that GF mice extract as much total energy from food as the other 2 groups. All 3 groups also have approximately equal energy expenditure, leading to neutral energy balance. (Bottom) During the light phase, GF and OligoMM12 mice have lower RER than conventional mice, but during the dark phase, OligoMM12 and conventional mice both have higher RER than GF mice. GF mice do not produce SCFAs, while OligoMM12 mice produce fewer SCFAs than conventional mice. Images were created with BioRender.com. GF, germ-free; RER, respiratory exchange ratio, SCFA, short-chain fatty acid.

The gut microbiome affects how its host uses energy [1]. Research into this topic often compares germ-free (GF) mice—mice that are born in a sterile environment and are never exposed to bacteria—to conventionally colonized, or specific-pathogen-free (SPF), mice. However, SPF mice may have very different gut microbiomes depending on the facility in which they are born and raised. For this reason, investigators are increasingly using gnotobiotic mice, i.e., mice with simple but well-defined microbial communities that can be replicated across facilities. An example of such a gnotobiotic model is the OligoMM12 mouse, whose microbiome consists of 12 cultivable bacterial strains representing 5 major bacterial phyla of the mouse gut microbiome [2]. While the effect of defined microbial communities on immune system development, inflammation, and host defense have been extensively characterized [3–5], their impact on host metabolism remains poorly understood. In a new study in *PLOS Biology*, Hoces and colleagues [6] thoroughly compared OligoMM12, GF, and SPF mice to determine the ways in which colonization with OligoMM12 does or does not recapitulate the properties of a conventional microbiome (**Fig 1**). A key technological innovation in this study was the use of an isolator-housed metabolic cage system. Using this setup, the authors were able to continuously monitor food intake, water consumption, temperature, and the levels of carbon dioxide, oxygen, and hydrogen gases under conditions of defined microbial colonization.

The authors made several interesting discoveries related to systemic metabolism. At first glance, GF mice weighed more than OligoMM12 and SPF mice, but this difference disappeared after subtracting the weight of the cecum, a section of the gastrointestinal tract between the small and large intestine, from total body mass. Cecum size dramatically expands in GF mice, probably due to intestinal osmosis and decreased intestinal motility [7,8]. Subtracting the weight of the cecum substantially changes the conclusion about body weight differences between the 3 groups; this highlights the importance of properly accounting for the massively enlarged cecum in microbiome-depleted mice.

Another notable difference in body composition is that OligoMM12 mice have greater fat mass than either GF or SPF mice. To further investigate this relationship between microbiome status and fat storage, the authors leveraged their isolator-housed metabolic cage system. Through indirect calorimetry (which relies on measuring carbon dioxide and oxygen levels), the authors found that all 3 groups had equivalent energy expenditure. GF mice had greater energy intake than the other 2 groups (they ate more food), but they also had greater energy loss (they produced more feces with lower fecal energy density), such that their increase in energy intake was perfectly compensated by increased fecal energy loss (**Fig 1**). In all 3 groups, energy extraction was approximately equal to energy expenditure, leading the authors to conclude that the increase in fat mass in OligoMM12 mice is likely not explained by an excess of calories being converted to fat.

Rather, the authors hypothesized that the increased fat mass might be due to different utilization of calories. In support of this hypothesis, all 3 groups had different patterns in their respiratory exchange ratio (RER)—a metric that quantifies whether fat or carbohydrates are being used for energy production—over the course of the day. There were also differences over the course of the day in levels of hepatic glycogen stores, hydrogen gas production, and levels of short-chain fatty acids (SCFAs) (the latter 2 being indicators of microbial metabolism), further supporting the idea that the 3 groups utilize the same caloric intake in different ways.

The authors continued their investigation of time-of-day differences in host metabolism by performing metabolomics of the liver and blood collected during the day and during the night. They found that both during the light and dark phases, the metabolomes of OligoMM12 mice were consistently more similar to those of GF mice than SPF mice. This finding suggests that this minimal microbiome is unable to fully reproduce the effects of a conventional microbiome on the host metabolome.

The study highlights several observations with broader implications for the investigation of microbiome effects on systemic metabolism. For instance, the authors discovered that EchoMRI is not reliable for measuring fat mass in GF mice. EchoMRI is an instrument that uses nuclear magnetic resonance to measure the percentage of body mass that is fat mass, lean mass, or water mass. The EchoMRI inaccuracy is related to GF mice having very large ceca. The authors discovered that, in GF mice only, estimates of fat mass before and after removal of the cecum were poorly correlated. They speculated that this was due to inconsistent assignment of cecal content as fat or water mass. In lieu of EchoMRI, the authors instead dissected and weighed various fat depots in order to estimate overall fat mass. This finding serves as a word of caution against using EchoMRI to quantify fat mass in animals with different microbiome status.

Another surprising finding was that GF mice had lower fecal energy density than the other 2 groups. Intuitively, one would expect GF mice to have higher fecal energy density because they lack microbes to help extract calories from food. However, the authors speculated that the lower fecal energy density is because microbial biomass itself contains calories. In support of this hypothesis, the authors calculated that the energy content of microbes in stool is approximately equal to the difference in fecal energy density between GF mice and the other 2 groups.

Along with a previous report [9], this study demonstrates the feasibility of multiday metabolic monitoring under gnotobiotic conditions. Because the monitoring was continuous, the authors were able to detect interesting time-of-day differences between groups. Such observations promote continued investigation into how microbiome complexity influences circadian biology [10]. One specific extension of this work is asking how the circadian system of OligoMM12 mice compares to mice with other reduced-complexity microbiomes, such as monocolonized mice or mice colonized with the altered Schaedler flora. The study also paves the way for the systematic investigation of commensal species that regulate the extraction and utilization of calories from food.

In summary, Hoces and colleagues have added to our understanding of the ways in which the metabolism of mice with a minimal microbiome does and does not resemble that of mice with a conventional microbiome. Although the precise mechanism regulating differences in energy storage between mice harboring the OligoMM12 community and a complex microbiota remains to be further elucidated, thoughtful studies such as this will pave the way toward increased reproducibility in microbiome experiments.

## Abbreviations

GF: germ-free
RER: respiratory exchange ratio
SCFA: short-chain fatty acid
SPF: specific-pathogen-free

## References

[1] Sonnenburg JL, Backhed F. Diet-microbiota interactions as moderators of human metabolism. Nature. 2016;535(7610):56–64. Epub 2016/07/08. doi: 10.1038/nature18846 ; PubMed Central PMCID: PMC5991619. 27383980 PMC5991619

[2] Brugiroux S, Beutler M, Pfann C, Garzetti D, Ruscheweyh HJ, Ring D, et al. Genome-guided design of a defined mouse microbiota that confers colonization resistance against Salmonella enterica serovar Typhimurium. Nat Microbiol. 2016;2:16215. Epub 2016/11/22. doi: 10.1038/nmicrobiol.2016.215 . 27869789

[3] Herp S, Brugiroux S, Garzetti D, Ring D, Jochum LM, Beutler M, et al. Mucispirillum schaedleri Antagonizes Salmonella Virulence to Protect Mice against Colitis. Cell Host Microbe. 2019;25(5):681–94 e8. Epub 2019/04/23. doi: 10.1016/j.chom.2019.03.004 . 31006637

[4] Geuking MB, Cahenzli J, Lawson MA, Ng DC, Slack E, Hapfelmeier S, et al. Intestinal bacterial colonization induces mutualistic regulatory T cell responses. Immunity. 2011;34(5):794–806. Epub 2011/05/21. doi: 10.1016/j.immuni.2011.03.021 . 21596591

[5] Tran HQ, Bretin A, Adeshirlarijaney A, Yeoh BS, Vijay-Kumar M, Zou J, et al. “Western Diet”-Induced Adipose Inflammation Requires a Complex Gut Microbiota. Cell Mol Gastroenterol Hepatol. 2020;9(2):313–33. Epub 2019/10/09. doi: 10.1016/j.jcmgh.2019.09.009 ; PubMed Central PMCID: PMC6957826. 31593782 PMC6957826

[6] Hoces D, Lan J, Wenfei S, Geiser T, Stäubli M, Cappio Barazzone E, et al. Metabolic reconstitution of germ-free mice by a gnotobiotic microbiota varies over the circadian cycle. PLOS Biol. 2022; 20(9):e3001743. doi: 10.1371/journal.pbio.3001743 36126044 PMC9488797

[7] Bayer F, Ascher S, Pontarollo G, Reinhardt C. Antibiotic Treatment Protocols and Germ-Free Mouse Models in Vascular Research. Front Immunol. 2019;10:2174. Epub 2019/10/02. doi: 10.3389/fimmu.2019.02174 ; PubMed Central PMCID: PMC6751252. 31572384 PMC6751252

[8] Nicklas W, Keubler L, Bleich A. Maintaining and Monitoring the Defined Microbiota Status of Gnotobiotic Rodents. ILAR J. 2015;56(2):241–9. Epub 2015/09/02. doi: 10.1093/ilar/ilv029 . 26323633

[9] Halatchev IG O’Donnell D, Hibberd MC, Gordon JI. Applying indirect open-circuit calorimetry to study energy expenditure in gnotobiotic mice harboring different human gut microbial communities. Microbiome. 2019;7(1):158. Epub 2019/12/14. doi: 10.1186/s40168-019-0769-4 ; PubMed Central PMCID: PMC6909537. 31831058 PMC6909537

[10] Litichevskiy L, Thaiss CA. The Oscillating Gut Microbiome and Its Effects on Host Circadian Biology. Annu Rev Nutr. 2022. Epub 2022/05/17. doi: 10.1146/annurev-nutr-062320-111321 . 35576592

